# Application of CRISPR/Cas9 Technology to HBV

**DOI:** 10.3390/ijms161125950

**Published:** 2015-11-02

**Authors:** Guigao Lin, Kuo Zhang, Jinming Li

**Affiliations:** National Center for Clinical Laboratories, Beijing Hospital, Beijing 100730, China; linguigao1982@163.com (G.L.); sarahkuo@163.com (K.Z.)

**Keywords:** CRISPR/Cas9, HBV, cccDNA, antiviral

## Abstract

More than 240 million people around the world are chronically infected with hepatitis B virus (HBV). Nucleos(t)ide analogs and interferon are the only two families of drugs to treat HBV currently. However, none of these anti-virals directly target the stable nuclear covalently closed circular DNA (cccDNA), which acts as a transcription template for viral mRNA and pre-genomic RNA synthesis and secures virus persistence. Thus, the fact that only a small number of patients treated achieve sustained viral response (SVR) or cure, highlights the need for new therapies against HBV. The clustered regularly interspaced short palindromic repeats (*CRISPR*)/*Cas9* gene editing system can specifically target the conserved regions of the HBV genome. This results in robust viral suppression and provides a promising tool for eradicating the virus. In this review, we discuss the function and application of the CRISPR/Cas9 system as a novel therapy for HBV.

## 1. Introduction

**Figure 1:**
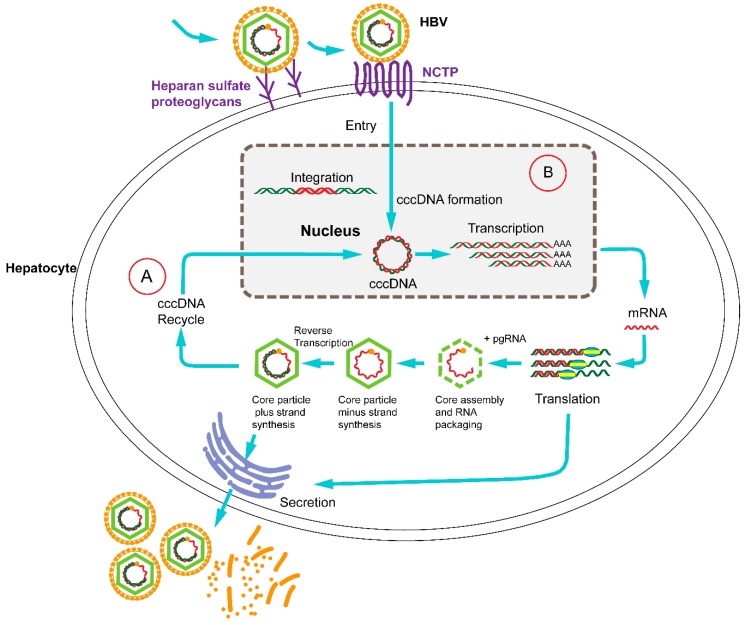
The life cycle of hepatitis B virus (HBV) with therapeutic targets. HBV binds to surface receptors and enters the hepatocyte. Viral particles migrate to the cell nucleus, where the HBV genomes are converted to a covalently closed circular (cccDNA) that serves as a template for viral transcription. The translation of viral mRNA in the cytoplasm results in the production of the core (C), polymerase (P), surface (S) and hepatitis B virus X (HBx) proteins. Next, genomic viral RNA is packaged into the progeny viral capsids. The core particle can either be encapsulated and secreted from the hepatocyte or be reimported into the nucleus for transformation to cccDNA. Clustered regularly interspaced short palindromic repeats (CRISPR)/Cas9-directed disruption of the HBV life cycle can target (A) or (B) the entry receptor sodium taurocholate co-transporting polypeptide (NTCP) which is necessary for the HBV life cycle.

The World Health Organization estimates that 240 million persons are chronically infected with hepatitis B virus (HBV), a hepatotropic DNA virus which replicates by reverse transcription [1,2]. Chronically infected individuals are at an increased risk for liver cirrhosis and hepatocellular carcinoma [3]. Currently, IFN-α and five oral nucleos(t)ide analogs (NUCs) are used for the treatment of chronic hepatitis B [4]. NUCs inhibit the synthesis of viral DNA from pregenomic RNA by targeting the multifunctional reverse transcriptase, named P protein. NUCs can effectively reduce viral DNA level by multiple logs; however, virus reactivation often occurs within weeks to months after treatment withdrawal [5,6]. In consequence, life-long of NUC therapy is usually required [7]. Additionally, long-term NUC treatment may lead to the selection of resistance-conferring mutations [8]. α interferons (IFN-α) has the advantage of a much shorter treatment period (6 months to 1 year) and without the risk for mutant selection. The main disadvantages of IFN-α are the injection-related adverse effects and the fact that only a small group of patients are eligible for interferon therapy and less than 10% of them achieve a sustained response [9]. The ultimate goal of chronic HBV therapy should involve the full elimination of all viral DNA from the body. Since a refractory intracellular HBV replication involves an intermediate which is termed covalently closed circular (ccc) DNA, current treatments rarely achieve a cure [10]. The highly stable HBV cccDNA, which is generated from the relaxed circular (RC) HBV DNA genome in the infected hepatocytes, acts as the template for viral mRNA and pre-genomic RNA synthesis, and in consequence gives rise to the new virions [11] (Figure 1).

To achieve significant rates of sustained virological responses or cure, new therapies with alternative mechanisms of action against HBV are now under development [4]. New investigational agents fall into two categories: direct-acting and host-targeting HBV inhibitors. Direct-acting antivirals target critical steps of the HBV life cycle and include HBsAg inhibitors, capsid inhibitors, RNaseH inhibitors, DNA cleavage enzymes, and siRNA-acting drugs [6]. On the other hand, host-targeting antivirals aim at targets involved in HBV entry and secretion and/or restore anti-virus immune responses [6]. For completely eradicating HBV, the virus cccDNA intermediates must be destroyed [12]. Novel nuclease-based gene targeting technologies, such as zinc finger nucleases (ZFNs), transcription activator-like effector nucleases (TALENs), and clustered regularly interspaced short palindromic repeats (CRISPR)/Cas9 system, have been harnessed to disrupt HBV genome with favorable effects [13,14,15,16,17,18,19,20,21,22,23]. These sequence-specific nucleases can create double-stranded breaks (DSBs) at specific DNA locations, which may stimulate error-prone nonhomologous end joining (NHEJ) pathway in the absence of repair templates, leading to the formation of insertions and deletions (indels). The indels at the DSB site can result in frameshifts and/or the production of premature stop codons, which disrupt the open reading frame (ORF) in the target gene. Given the highly difficult design and costly preparation of the ZFNs and TALENs, the CRISPR/Cas9 system has rapidly developed as a simpler and more versatile gene editing technology [24]. Here, we review the function and application of CRISPR/Cas9 as a novel therapy for HBV.

## 2. The Emergence of CRISPR/Cas9: From Bacterial Immune System to Versatile Genome Editing Tool

**Figure 2:**
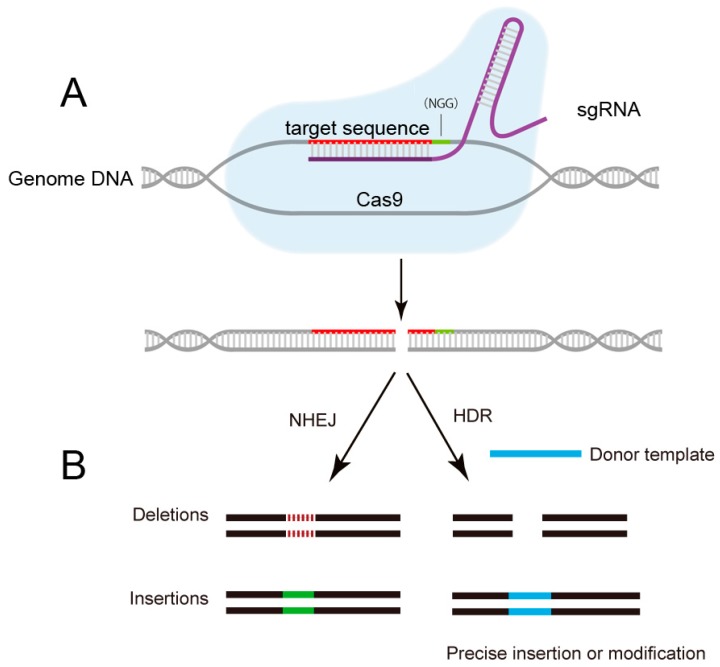
Schematic of CRISPR/Cas9 mediated-genome editing. (**A**) The sgRNA guides the Cas9 protein to cut specific DNA sequence by recognizing the protospacer-adjacent motif (PAM) sequence and a complementary target sequence; (**B**) The breaks induced by Cas9 are repaired either through homology-directed repair (HDR) or non-homologous end joining (NHEJ), results in precise gene editing with insertions or deletions (indels), respectively. NGG: PAM sequence.

The CRISPR system was originally found to be a bacterial adaptive immune defense system against invading plasmids and phages [25]. Ribonucleoprotein complexes formed with CRISPR RNAs (crRNAs), trans-activating crRNA (tracrRNA), and Cas proteins can perform crRNA-guided recognition and degradation of foreign nucleic acids [26]. The Cas protein-mediated DNA cleavage requires a complementarity between the target sequence and the crRNA, and the presence of a proto spacer adjacent motif (PAM). Later, the type II CRISPR/Cas system from *Streptococcus pyogenes* was further engineered with the development of a chimeric single-guide RNA (sgRNA) which consists of a fusion of crRNA/tracerRNA and a Cas9 protein [27] (Figure 2). Importantly, the sgRNA and Cas9 protein are sufficient for induction of targeted DNA binding and cleavage in a variety of systems, including cultured human cells, rats, mice, *Caenorhabditis elegans*, zebrafish, *Drosophila*, bacteria, *Arabidopsis thaliana*, and others [28,29,30,31,32,33,34,35,36]. This two-component system can be utilized by targeting to any DNA sequences with the PAM form, making it a highly versatile tool for various applications.

## 3. CRISPR/Cas9 Target HBV cccDNA and Inhibit HBV Replication

**Figure 3:**
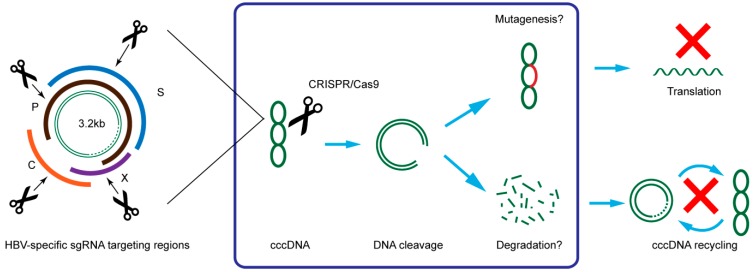
Illustration of HBV targeting strategy and possible mechanism of CRISPR/Cas9 in suppression of HBV. C: core; P: polymerase; S: surface; X: HBx.

The HBV genome has four long open reading frames (surface, core, polymerase, and X protein) which are translated into only seven proteins highly important for viral replication [37]. In theory, targeting any one of the seven proteins would likely be sufficient to suppress viral gene expression and replication (Figure 3).

There are several examples of targeting HBV genome with varying degrees of success by CRISPR/Cas9 system (Table 1). Lin *et al.* first reported that the CRISPR/Cas9 system could be used to disrupt the HBV genome both *in vitro* and *in vivo* [15]. They showed that HBV-specific Cas9/sgRNA combinations were able to significantly reduce the production of HBV core and HBsAg when Cas9 and a HBV expression plasmid were co-transfected into Huh7 hepatocyte-derived cellular carcinoma cells. In addition, this system could efficiently reduce levels of intrahepatic HBV-expressing vectors and the serum levels of HBsAg in an HBV hydrodynamics-mouse model. Using lentiviral transduction of Cas9 and HBV-specific gRNAs, Kennedy *et al.* extended these findings by demonstrating effective inhibition of HBV DNA production and cccDNA accumulation for *in vitro* models of both chronic HBV infection (HepAD38 cells) and *de novo* infection (HepaRG cells) [16]. The CRISPR/Cas9 system suppressed total HBV viral DNA levels by up to ~1000-fold and cccDNA levels by up to ~10-fold. Seeger and Sohn demonstrated that HBV infections could be inhibited up to eightfold by HBV-specific guide RNAs in sodium taurocholate cotransporting polypeptide (NTCP) expressing HepG2 cells [17]. In another study, Liu *et al.* reported that HBV-specific gRNA/Cas9 could inhibit the replication of HBV of different genotypes both *in vitro* and *in vivo*, which was due to clearance and error prone repair of viral DNA templates [18]. Zhen *et al.* targeted the surface ORF, both in HepG2.2.15 cells and an *in vivo* hydrodynamics-mouse model [19]. The HBsAg levels in the culture supernatants and mouse serum were lowered by CRISPR/Cas9 treating. The system could also effectively inhibit HBV DNA levels and HBsAg expression in mouse livers. Dong *et al.* demonstrated that the CRISPR/Cas system could be used for inhibiting intracellular cccDNA and viral replication in precccDNA-transfected Huh7 cells and in a new mouse model carrying HBV cccDNA [20]. Ramanan *et al.* showed that sgRNAs targeting conserved regions of HBV cause strong inhibition of virus replication both *in vitro* and *in vivo*, and extended this antiviral activity to virus isolated from patients [21]. Upon continuous expression of Cas9/sgRNA, the group demonstrated a sharp decline of cccDNA and HBV proteins in a *de novo* infection model. Wang *et al.* applied dual gRNAs to guided CRISPR/Cas9 system to inactivate HBV of genotypes A–D *in vitro* [22]. In the most recent study of HBV and CRISPR, Karimova *et al.* demonstrated that an improved CRISPR/Cas9 nickase system can disrupt both HBV cccDNA and integrated HBV sequences in HeLa and HEK293 cell lines [23]. Also, by targeting S- or X-ORFs, they successfully inhibit HBsAg expression in both chronically and novel *de novo* infected human hepatoma cell lines. In summary, these studies have demonstrated the usefulness of the CRISPR/Cas9 system in destroying HBV cccDNA both *in vitro* and *in vivo*, and shown the therapeutic potential of CRISPR/Cas9 in acute and chronic HBV infection.

**Table 1:** Application of CRISPR/Cas9 to HBV.

Target	HBV Infection Model	Results	Reference
P1, S1, XCp, and PS2 ORFs	Huh7	Reduction in HBsAg level in medium	Lin *et al.* [15]
	HBV hydrodynamics-mouse model	Reduction in HBsAg level in serum	Lin *et al.* [15]
P, S, and C ORFs	HepAD38 and HepaRG	Reduction in viral DNA and cccDNA levels. Reduction in HBsAg and HBeAg level in medium	Kennedy *et al.* [16]
ENII-CP/X and Pre-C ORFs	HepG2 with HBV receptor NTCP	Eight-fold inhibition of HBV infection	Seeger and Sohn [17]
P, S, X and C ORFs	HepG2	Reduction of intracellular HBV replication intermediates and extracellular virion DNA	Liu *et al.* [18]
	HBV hydrodynamics-mouse model	Reduction in HBsAg and HBeAg level in serum and the expression of HBcAg in liver	Liu *et al.* [18]
P, S, X and C ORFs	HepG2.2.15	Reduction in HBsAg level in medium and intracellular cccDNA	Zhen *et al.* [19]
	HBV hydrodynamics-mouse model	Reduction in HBsAg level in serum	Zhen *et al.* [19]
X/L and X ORFs	Huh7	Reduction in HBsAg and HBeAg level in medium and intracellular cccDNA	Dong *et al.* [20]
	HepG2.2.15	Reduction in HBsAg level in medium	Dong *et al.* [20]
	HBV hydrodynamics-mouse model carrying cccDNA	Reduction in HBsAg and HBeAg level in serum and intrahepatic cccDNA	Dong *et al.* [20]
P, S, X and C ORFs	HepG2 with HBV receptor NTCP	Reduction in HBsAg, HBV DNA, 3.5kb RNA and cccDNA levels in culture medium	Ramanan *et al.* [21]
	HepG2.2.15	Reduction in HBV DNA and cccDNA levels	Ramanan *et al.* [21]
	HBV hydrodynamics-mouse model	Reduction in HBsAg and viral DNA level in serum	Ramanan *et al.* [21]
P, S, X and C ORFs	HuH-7	Reduction in HBsAg and HBeAg level in medium	Wang *et al.* [22]
	HepAD38	Reduction in HBsAg, HBeAg, HBV DNA, and cccDNA levels in culture medium	Wang *et al.* [22]
S and X ORFs	HepG2.2.15 and HepG2-H1.3	Significant reduction in HBsAg level in medium	Karimova *et al.* [23]
	HepG2 ^hNTCP^	Significant reduction in HBsAg level in medium	Karimova *et al.* [23]

P: polymerase; S: surface; X: HBx; C: core; ORF: open reading frame; XCp: X core promotor; cccDNA: covalently closed circular DNA; L: large surface protein; PS2: pre-S2; CP: core promoter; ENII-CP: enhancer II and core promoter.

## 4. The Limitations of the CRISPR/Cas9 Technology as a Novel Therapeutic for HBV

Current studies provide a proof of concept, but there are significant issues that need to be addressed before the translation of CRISPR/Cas9 systems to clinical HBV treatment.

The greatest concern is the ability to eradicate all viruses. However, the best result of HBV cleavage using CRISPR/Cas9, achieved by Ramanan *et al.*, is reduction of cccDNA by about 92% in culture cell [19]. A potential challenge is that HBV DNA can be found in various tissues outside the liver and a range of cell lines are permissive for HBV replication [38,39,40]. For eradication of HBV, it is essential to deliver the nucleases to every last infected cell in hepatic and extrahepatic viral reservoirs. To achieve sustained anti-HBV activity *in vivo*, an efficient delivery vehicle should be used. Due to the low immune potential, non-integrating nature, and high infection efficiency, recombinant adeno-associated viral vectors (rAAVs) is currently the best choice for delivering CRISPR/Cas9. Nevertheless, the total length of the humanized *Cas9* gene (~4.2 kb), sgRNA and the regulatory elements surpasses the ~4.5 kb cargo size of rAAV [41]. This packaging obstacle can be solved by taking advantage of a split-Cas9 system [42] or by using a smaller Cas9 orthologs, such as SaCas9 from *Staphylococcus aureus*, which is >1 kb shorter [41].

The second concern is the potential off-target effects with CRISPR/Cas9 system [43]. Only one of the nine studies conducted next-generation sequencing at limited potential off-target locations for HBV specific sgRNAs [15,16,17,18,19,20,21,22,23]. While Cas9-directed cleavage has not been detected within the human genome at sites of homology to viral Cas9 target, an extensive genome-wide off-target analysis is warrant. The data published previously showed that significant off-target activity of CRISPR/Cas9 system does occur at a high rate, even for sgRNAs that have mismatches up to five nucleotides [43]. Several approaches have been developed to reduce off-target effects: (i) The “paired nicking” strategy [44]. By using two spaced gRNAs and a mutated Cas9 (Cas9n) with single-strand DNA cleavage capacity, a nick instead of DSBs can be produced on both DNA strands. With the opposite intact strand as a template, nicks would be further repaired precisely by the cell machine. The paired sgRNAs and Cas9n was reported to reduce off-target effect by 1500-fold comparing to the wild-type Cas9; (ii) Truncate sgRNAs at the 5′ end of the complementary targeting sequence. Fu *et al.* demonstrated that shorter sgRNAs with 17 or 18 nucleotides can improve target specificity by more than 5000-fold without reducing the on-target efficiency [45]; (iii) Another variant Cas9 (fCas9) was engineered by fusing catalytically inactive Cas9 to Fok I nuclease [46]. The cleavage induced by fCas9 requires that each part of the dimmer binding to DNA. Using the fCas9 system, a specificity of 140-fold higher than typical Cas9 was achieved in human cell by increasing the number of targeting bases [46]. However, these approaches remain less efficient.

The third concern is selecting proper target sites in the HBV genome. The major problem of Nucleos(t)ide analog treatment is the emergence of therapy-resistant HBV variants. Given that the CRISPR/Cas9 system relies on precise sequence recognition, if the viral load is sufficiently high, viral genomes with *de novo* mutations that cause loss of the CRISPR/Cas9 recognition site will be selected for, and would provide a therapy-resistant pool of viruses able to re-establish infection. Thus the targeting sequence should avoid overlapping with regions such as (Y)-methionine (M)-aspartic acid (D)-aspartic acid (D) (YMDD) motif. Importantly, HBV is classified into eight genotypes A–H since the high sequence divergence. Targeting to highly conserved sequences among different viral genotypes and using multiple CRISPR/Cas9 constructs simultaneously, are good strategies for minimising the problem of existing and *de novo* mutations.

The last concern is the integrated linearised HBV DNA, which may be cut by the CRISPR/Cas9. The integration of subgenomic HBV DNA fragments into host genome is common in patients with hepatocellular carcinoma or chronic infection [47]. The cleavage of integrated viral DNA can also leads to indels in the host genome, which may potentially disrupt the host gene function. The unwanted adverse effect should be carefully evaluated.

## 5. Potential Gene Editing Target for HBV Therapy with CRISPR/Cas9

Novel Genome editing technologies have been recently used as weapons against several human viruses [48,49]. In the field of curative HIV research, studies showed that CD4^+^ T cells with chemokine (CC motif) receptor 5 (CCR5)-disrupted by nucleases are resistance to HIV-1 infection [50,51]. A major breakthrough is that ZFNs which cleave CCR5 have entered phase I clinical trials [52]. Sodium taurocholate co-transporting polypeptide, which mediates the transport of bile acids and other molecules from portal circulation, was recently identified as an entry receptor of HBV and HDV [53]. One category of the indirect anti-HBV agents in development are entry inhibitors, which targeting the HBV receptor NTCP [54]. With the encouraging result of nucleases based *CCR5* gene therapy, it stands to reason that using CRISPR/Cas9 to disrupt NTCP expression may block the entry of HBV and prevent the spread of viral infection. Again, although no reports of serious diseases associated with defects in the NTCP gene have been published yet, the potential alteration of gene function and toxicity of NTCP disruption should be evaluated.

## 6. Conclusions

The CRISPR/Cas9 technology has tremendously advanced our ability to alter the genome and also brought about a new age of gene therapies to treat diseases. Although still in a proof-of-concept stage, CRISPR/Cas9 system can be used to inhibit HBV replication and gene expression both *in vitro* and *in vivo*, and may constitute a new therapeutic approach for HBV infection. A combination of CRISPR/Cas9-based therapy and RT inhibitors currently used may achieve the highest rates of viral response. While many obstacles remain, including safety and delivery efficiency of the system, it is clear that CRISPR/Cas9 technologies offer great promise for the cure of chronic HBV infection.
